# Retention of Neutralizing Response against SARS-CoV-2 Omicron Variant in Sputnik V-Vaccinated Individuals

**DOI:** 10.3390/vaccines10050817

**Published:** 2022-05-21

**Authors:** Daniele Lapa, Daria M. Grousova, Giulia Matusali, Silvia Meschi, Francesca Colavita, Aurora Bettini, Giulia Gramigna, Massimo Francalancia, Anna Rosa Garbuglia, Enrico Girardi, Vincenzo Puro, Andrea Antinori, Anna V. Kovyrshina, Inna V. Dolzhikova, Dmitry V. Shcheblyakov, Amir I. Tukhvatulin, Olga V. Zubkova, Vladimir A. Gushchin, Denis Y. Logunov, Boris S. Naroditsky, Francesco Vaia, Alexander L. Gintsburg

**Affiliations:** 1 INMI “National Institute of Infectious Diseases” Lazzaro Spallanzani, IRCCS, 00149 Rome, Italy; daniele.lapa@inmi.it (D.L.); giulia.matusali@inmi.it (G.M.); silvia.meschi@inmi.it (S.M.); francesca.colavita@inmi.it (F.C.); aurora.bettini@inmi.it (A.B.); giulia.gramigna@inmi.it (G.G.); massimo.francalancia@inmi.it (M.F.); enrico.girardi@inmi.it (E.G.); vincenzo.puro@inmi.it (V.P.); andrea.antinori@inmi.it (A.A.); francesco.vaia@inmi.it (F.V.); 2 FSBI “National Research Centre for Epidemiology and Microbiology Named after Honorary Academician N F Gamaleya” of the Ministry of Health of the Russian Federation, 123098 Moscow, Russia; grousova@gamaleya.org (D.M.G.); kovyrshina@gamaleya.org (A.V.K.); dolzhikova@gamaleya.org (I.V.D.); scheblyakov@gamaleya.org (D.V.S.); amir_tukhvatulin@gamaleya.org (A.I.T.); zubkova@gamaleya.org (O.V.Z.); vladimir.a.gushchin@gamaleya.org (V.A.G.); logunov@gamaleya.org (D.Y.L.); bsnar@gamaleya.org (B.S.N.); gintsburg@gamaleya.org (A.L.G.); 3 Federal State Autonomous Educational Institution of Higher Education, I.M. Sechenov First Moscow State Medical University (Sechenov University), Ministry of Health of the Russian Federation, 119435 Moscow, Russia

**Keywords:** SARS-CoV-2, vaccine, antibody response, Omicron, neutralization

## Abstract

The new Omicron variant of SARS-CoV-2, first identified in November 2021, is rapidly spreading all around the world. Omicron has become the dominant variant of SARS-CoV-2. There are many ongoing studies evaluating the effectiveness of existing vaccines. Studies on the neutralizing activity of vaccinated sera against the Omicron variant are currently being carried out in many laboratories. In this study, we have shown the neutralizing activity of sera against the SARS-CoV-2 Omicron variant compared to the reference Wuhan D614G variant in individuals vaccinated with two doses of Sputnik V up to 6 months after vaccination and in individuals who experienced SARS-CoV-2 infection either before or after vaccination. As a control to our study we also measured neutralizing antibody titers in individuals vaccinated with two doses of BNT162b2. The decrease in NtAb titers to the Omicron variant was 8.1-fold for the group of Sputnik V-vaccinated individuals. When the samples were stratified for the time period after vaccination, a 7.6-fold or 8.8-fold decrease in NtAb titers was noticed after up to 3 and 3-to-6 months after vaccination. We observed a 6.7- and 5-fold decrease in Sputnik V-vaccinated individuals experiencing asymptomatic or symptomatic infection, respectively. These results highlight the observation that the decrease in NtAb to the SARS-CoV-2 Omicron variant compared to the Wuhan variant occurs for different COVID-19 vaccines in use, with some showing no neutralization at all, confirming the necessity of a third booster vaccination.

## 1. Introduction

As of 14 April 2022, the Omicron variant has been confirmed in at least 150 countries around the world [1]. From 3 January 2022 to 9 January 2022, the Omicron variant accounted for roughly 62.5 percent of all SARS-CoV-2 sequences available on the GISAID database [2].

Today, the effectiveness of existing vaccines against the new Omicron variant is the question of high priority. Around the world, studies are being carried out to analyze the neutralization activity of the sera of vaccinated people against the Omicron variant.

It was shown that the sera from vaccinated individuals with two doses of BNT162b2 or mRNA-1273 has a 22–30-fold decrease in neutralizing activity against the Omicron variant compared with the wild type virus [3,4]. A similar decrease in neutralizing activity was shown in other studies comparing two vaccines: ChAdOx1-nCoV-19 and BNT162b2 [4,5]. A study was conducted in China comparing the effectiveness of the BNT162b2 and CoronaVac vaccines. According to the results of the study, no neutralizing antibodies against the Omicron variant were shown in individuals vaccinated with CoronaVac [6].

However, many studies have shown that the administration of three doses of vaccine or a combination of the disease and vaccination significantly increases the titer of virus-neutralizing antibodies to the Omicron variant [7,8]. Analysis of the sera of individuals only vaccinated with BNT162b2, mRNA-1273 or Ad26.COV2.S has shown the decrease in neutralizing activity against the Omicron variant from 23- to 122-fold [9,10]. Preliminary results on Sputnik V showed a decrease in neutralizing antibody titers from 4- to 13-fold in the case of a combination of infection and vaccination [11].

In this study, we have shown the neutralizing activity of sera against the Omicron variant in individuals vaccinated with two doses of Sputnik V or BNT162b2 at different time points after vaccination and in Sputnik V-vaccinated individuals with a history of COVID-19.

## 2. Materials and Methods 

### 2.1. Ethics Statement

The study was conducted according to the guidelines of the Declaration of Helsinki, and approved by Gamaleya NRCEM Local Ethics Committee (Protocol No. 17 of 3 December 2021).

### 2.2. Samples for Analysis

Serum samples from 31 Sputnik V-vaccinated individuals were collected at 1 week to 6 months from the second dose. Moreover, serum samples from Sputnik V vaccinees with a history of either asymptomatic (*n* = 8) or mild (*n* = 12) SARS-CoV-2 infection were also analysed. Sera from 17 BNT162b2 vaccinated health care workers were collected at two weeks, 3 months and 6 months from the second vaccine dose (*n* = 51) in the context of a longitudinal study on SARS CoV2 vaccination approved by the ethical committee at National Institute for Infectious Diseases L. Spallanzani IRCCS (INMI), decision n.297/2021. Peripheral blood from COVID-19 convalescent individuals (*n* = 23) was collected at 1 to 3 months from symptom onset. Part of COVID-19 convalescent samples were plasma samples obtained by plasmapheresis. Tables S1–S3 report the age and gender characteristics of each group included in the present study. Serum/plasma samples were centrifuged at 1000× *g* for 10 min, aliquoted and stored at −80 °C until use. 

### 2.3. Receptor-Binding Domain (RBD)- and Nucleoprotein (N)-Specific IgG Evaluation 

Two commercial chemiluminescence microparticle antibody assays (ARCHITECT, Abbott Laboratories, Wiesbaden, Germany) were used, according to manufacturer’s protocols: the anti-nucleoprotein IgG and the SARS-CoV-2 IgG II kit, which detected antibodies against the RBD of SARS-CoV-2. Index values ≥ 1.4 and values ≥ 7.1 BAU/mL are considered positive for anti-N IgG and anti-RBD IgG, respectively.

### 2.4. SARS-CoV-2 Variants

As a Wuhan D614G reference strain, we used isolate B.1 (SARS-CoV-2/Human/ITA/PAVIA10734/2020, EVAG Ref-SKU 008V-04005, GISAID accession ID EPI_ISL_568579). The Omicron variant of SARS-CoV-2 was isolated from a nasopharyngeal swab of a traveler returning to Italy in December 2021 (hCoV-19/Italy/LAZ-INMI-2890/2021, GISAID accession ID EPI_ISL_7716384).

### 2.5. Cell Culture, Virus Isolation and Propagation 

Viral isolation was performed on Vero E6/TMPRSS2 (kindly provided by Dr. Oeda S., National Institute of Infectious Diseases, Tokyo, Japan). Initial passage, propagation and titration were performed on Vero E6 cells (ATCC CRL-1586). Cells were maintained in Minimal Essential Medium (MEM), containing 10% heat-inactivated fetal bovine serum (Corning), L-glutamine (Corning) and penicillin/streptomycin solution (Corning). 

Virus titer was determined by limiting dilution assay and residual infectivity was expressed as 50% Tissue Culture Infective Dose (TCID50/mL) calculated according to the Reed and Muench method. All work with infectious SARSCoV-2 virus was performed under biosafety level 3 (BSL-3) conditions at INMI. 

### 2.6. Neutralization Assay 

Neutralizing antibodies against SARS-CoV-2 variants in sera/plasma samples were analyzed by microneutralization test. The assay was performed as described earlier [12], using B.1 and VOC B.1.1.529 as challenging viruses and using a starting sample dilution of 1:5. Briefly, serum/plasma samples, after heat inactivation (+56 °C for 30 min), were serially diluted in MEM supplemented with 2% HI-FBS with starting sample dilution at 1:5 with two-fold dilution and mixed with 100 TCID50 SARS-CoV-2 at 1:1 ratio (50 μL serum dilution and 50 μL virus suspension), and incubated at 37 °C for 30 min. After that, serum–virus complexes were transferred to Vero E6 cells in 96-well plates and incubated for 48 h for B.1. and for 72 h for B.1.1.529 (Omicron). The 90% cytopathic effect (CPE) was assessed visually, if even a slight damage to the monolayer (1–3 «plaques») was observed in the well. Wells with more damage to the monolayer (4 and more «plaques») were considered to have a manifestation of CPE. Neutralization titer was defined as the highest serum dilution with 90% CPE or without CPE in two replicable wells. To standardize the inter-assay procedures, positive control samples showing high and low neutralizing activity were included in each session.

### 2.7. Statistical Analysis 

Statistical analysis was performed in GraphPad Prism version 9.2.0 (GraphPad Software Inc., San Diego, CA, USA). For comparison of paired data, Wilcoxon test was used, for comparison of unpaired data—Mann–Whitney test. For correlation analysis, Spearman test was used.

## 3. Results

We carried out a study of the neutralizing activity of the blood sera of the Sputnik V-vaccinated individuals either naïve or with a history of SARS-CoV-2 infection (before or after vaccination) against the Omicron and Wuhan D614G variants of SARS-CoV-2. 

**Figure 1:**
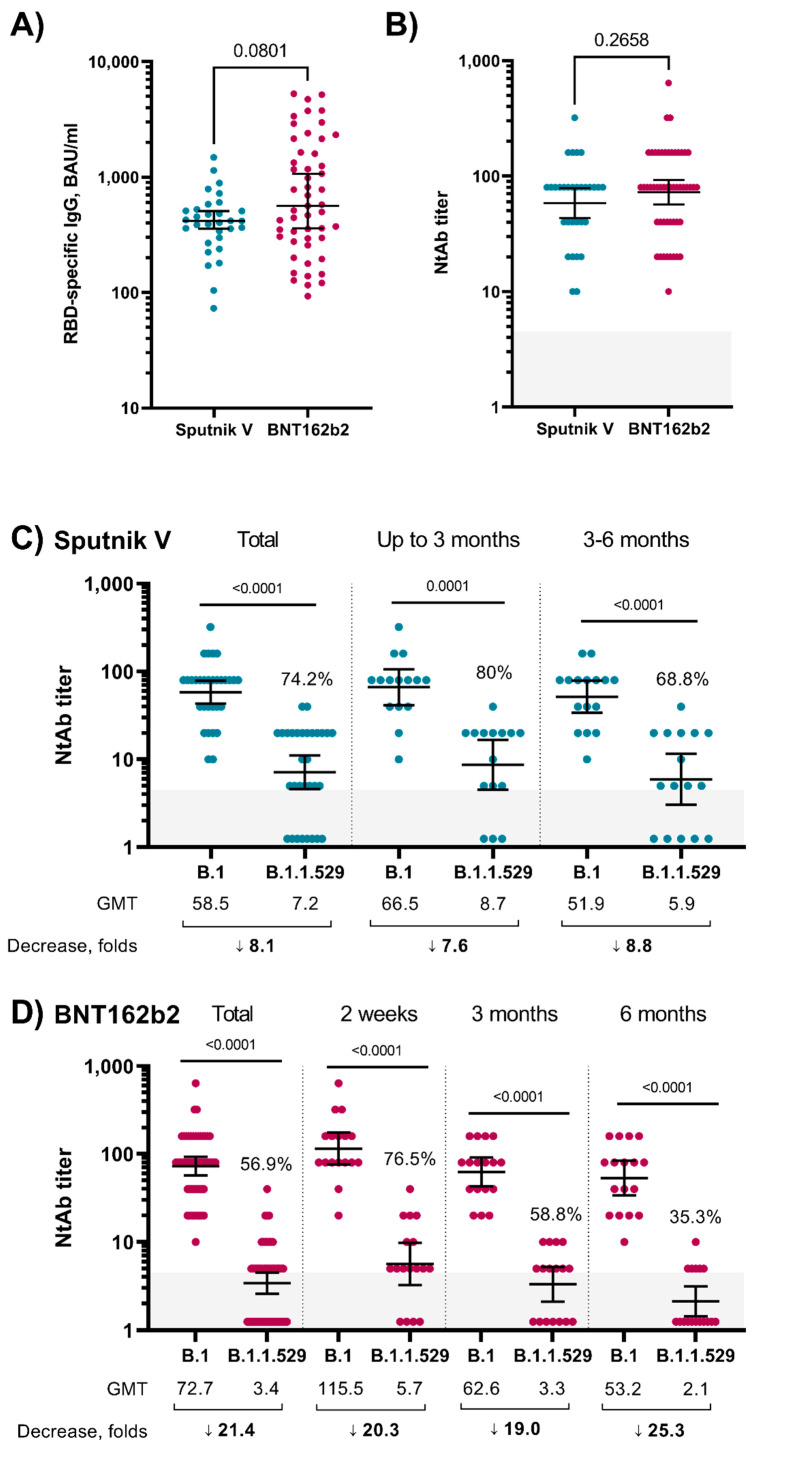
(**A**)—RBD-specific IgG titers in vaccinated individuals, median and 95% CI; (**B**)—NtAb titers against SARS-CoV-2 B.1 in vaccinated individuals, geometric mean titer and 95% CI, C, D—NtAb titers against SARS-CoV-2 B.1 (Wuhan D614G) and B.1.1.529 (Omicron) variants in Sputnik V- (**C**) and BNT162b2 (**D**)-vaccinated individuals, geometric mean titer and 95% CI, %—part of sera with detectable NtAb. *p*-value was determined by Wilcoxon test. Grey box—limit of detection. Values below the limit of detection were assigned a value of NtAb 1.25.

In the Sputnik V-vaccinated group with no history of COVID-19 (absence of antibodies to SARS-CoV-2 N-protein, no report of SARS-CoV-2 positive molecular test) we included individuals who were vaccinated with two doses of Sputnik V (*n* = 31; median and IQR of time after the second dose of the vaccine was 91 days (56–122)). The description of the groups is presented in Table S1. In addition, we analysed serum samples from individuals vaccinated with two doses of BNT162b2 (51 samples from 17 individuals, collected at 2 weeks, 3 months and 6 months from the second vaccine dose) and with no history of COVID-19 (the median and IQR of the time after the second dose were 90 days and 14–180 days, respectively, for BNT162b2) (Table S2). The analysis of RBD-specific antibodies showed that antigen-specific IgG was detected in all samples (Figure 1A), and the level of IgG between the Sputnik V and BNT162b2 groups was comparable (*p* = 0.0801, Mann–Whitney test). IgG dynamics were different in the groups: Sputnik V sera had stable IgG level in time, while samples from BNT162b2-vaccinated subjects showed a peak of the IgG response at 2 weeks with a significant decrease at the 3 and 6 month timepoints (Figure S1). The analysis of the neutralizing antibodies (NtAb) to the Wuhan D614G (B.1) variant also did not show statistically significant differences between the groups (Figure 1B, *p* = 0.2658, Mann–Whitney test). The correlation analysis of RBD-specific IgG and NtAb showed a significant correlation in both groups (Sputnik V: r = 0.69, *p* < 0.0001; BNT162b2: r = 0.56, *p* < 0.0001; Spearman test; Figure S2). The decrease in the NtAb level to the Omicron variant in comparison to the B.1 variant in the Sputnik V-vaccinated sera was 8.1-fold (GMT 58.5 vs. 7.2), and Omicron-specific NtAbs were detected in the blood sera of 74.2% of the Sputnik V vaccinees (Figure 1C). In the sera of individuals vaccinated with BNT162b2, we observed a 21.4-fold decrease in neutralizing activity (GMT 72.7 vs. 3.4) and 56.9% NtAb positive samples against Omicron (Figure 1D).

Next, we stratified the samples based on the time period after receiving the second dose of vaccines (Figure 1C,D). In the sera of individuals vaccinated with a second dose less than 3 months ago with Sputnik V (*n* = 15), the decrease in NtAb to the Omicron variant was 7.6-fold, and NtAbs to the Omicron variant were detected in 80% of the samples. For samples from individuals who received the second dose of Sputnik V 3–6 months ago (*n* = 16), the NtAb titers showed an 8.8-fold decrease (NtAbs were detected in 68.8% of samples) (Figure 1C). The analysis of the samples from individuals vaccinated with BNT162b2 (Figure 1D) showed that 2 weeks after the second dose, there was a 20.3-fold decrease in NtAbs to the Omicron variant (NtAbs to Omicron variant were detected in 76.5% of samples). In the subgroups of 3 and 6 months after the second dose, the decrease in the NtAb titer was 19- and 25.3-fold, respectively, while NtAbs to Omicron were detected in 58.8% and 35.3% samples, respectively (Figure 1D).

**Figure 2:**
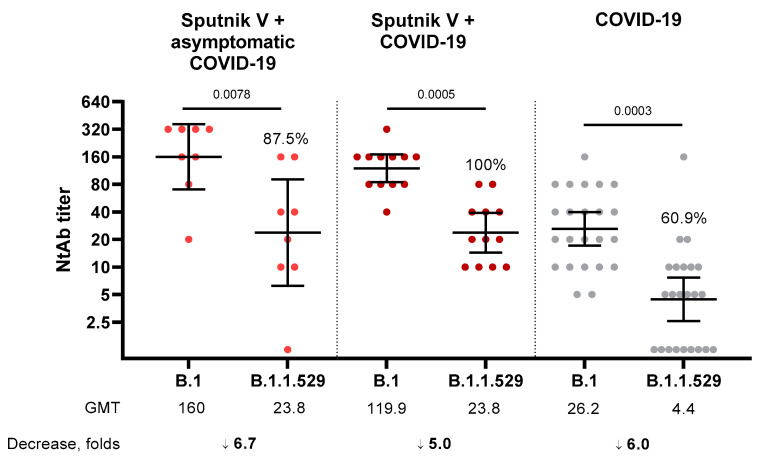
Titers of neutralizing antibodies in samples of individuals vaccinated with Sputnik V; vaccinated with Sputnik V after/before asymptomatic COVID-19; vaccinated with Sputnik V after/before mild COVID-19 and COVID-19 convalescents. The figure shows individual data for the studied samples, geometric mean and 95% CI. The figure shows the *p*-value (Wilcoxon test), % of individuals with detectable NtAb to the SARS-CoV-2, geometric mean and the level of NtAb decrease. Grey box—limit of detection. Values below the limit of detection were assigned a value of NtAb 1.25.

We also analyzed the NtAb level in the blood sera of individuals who were vaccinated with Sputnik V and underwent SARS-CoV-2 infection before or after the vaccination either in an asymptomatic (there is no history of COVID-19, but there are antibodies specific to the N-protein) or in symptomatic form (medical record of mild/moderate COVID-19) and in not vaccinated COVID-19 convalescents. In the sera of people vaccinated with Sputnik V and undergoing asymptomatic SARS-CoV-2 infection (*n* = 8) and symptomatic COVID-19 (*n* = 12), the decrease in NtAb titers against the Omicron variant was 6.7-fold (GMT 160 vs. 23.8, NtAbs to Omicron were detected in 87.5% of samples) and 5.0-fold (GMT 119.9 vs. 23.8, NtAbs to Omicron detected in 100% of samples), respectively. Analysis of the convalescent samples (*n* = 23) showed a 6.0-fold decrease to the Omicron variant with 60.9% responders (Figure 2).

## 4. Discussion

In this study we investigated the neutralizing activity of sera from two-dose Sputnik V- vaccinated individuals. For this purpose, we performed a live-virus neutralization assay with SARS-CoV-2 Wuhan (B.1) and Omicron (B.1.1.529) variants. As a control to our study we also assessed the ability of serum samples from BNT162b2 (two doses)-vaccinated health care workers to neutralize the Omicron variant. 

Previous reports on the ability of serum samples derived from Sputnik V vaccinees described an efficient neutralization of the B.1.1.7 variant, but a reduction against the B.1.351 VOC [13]. We found an 8.1-fold decrease in virus-neutralizing antibodies to the Omicron vs. the Wuhan-D614G variant in the group of Sputnik V-vaccinated subjects. Two recent studies also focusing on the Omicron BA.1 variants neutralizing response upon Sputnik V vaccination, showed variable fold decreases of NtAb titers when compared to a B.1 lineage SARS-CoV-2 reference strain [10,14]. The observed reduction in neutralizing response against BA.1 described here and in other reports strongly support the administration of a booster dose. Notably, the 21.4-fold reduction detected in our study among the BNT162b2-vaccinated group used as control group was in line with other recent publications [3,4,5,6]. Samples from BNT162b2-vaccinated individuals showed a peak of IgG response at 2 weeks with a significant decrease at the 3 and 6 month timepoints, while, in contrast to previous reports [14,15], Sputnik V sera showed stable IgG dynamics over time. Differences in the studied populations may have an impact on the results, and the samples here analysed for Sputnik V were not longitudinally collected in a follow-up designed trial. Nevertheless, this is an observational study and analysis of larger cohorts will be needed.

We divided each group of vaccinated sera into seven parts according to RBD-specific IgG (BAU/mL) quartiles (0–25%, >25–50%, >50–75%, >75–100%, 0–50%, >25–75% and >50–100%) and counted the decrease in NtAb and percent of sera with detectable NtAb (Table S4). We showed similar levels of neutralization drops among different groups, divided on the basis of quartiles (Table S4).

We also analyzed the neutralizing activity of sera of Sputnik V-vaccinated individuals with a history of mild or asymptomatic COVID-19. The decrease in NtAb titers to Omicron in the groups of Sputnik V-vaccinated COVID-19 convalescents, both mild and asymptomatic, was 5.0-fold and 6.7-fold, respectively. In both groups, the fold decrease in NtAb titers was lower than in vaccinated groups and GMT against the Omicron variant was 23.8, while GMT in vaccinated groups did not exceed 7.0. These data on hybrid immunity are in line with previously published data for other vaccines [16]. The decrease in NtAb in Sputnik V and Sputnik V + COVID-19 samples was similar to what was shown in samples from unvaccinated–COVID-19 convalescent individuals where a 6.0-fold decrease in the NtAb titer against Omicron was observed. However, taking into account that the initial GMT NtAb against B.1 in Sputnik V recipients was significantly higher compared to COVID-19 convalescents, a similar decrease in neutralizing activity against B.1.1.529 resulted in different percentages of individuals with detectable levels of NtAb against Omicron VOC. These results also provide evidence for Sputnik V that vaccination could be more efficient at providing immune protection against emerging variants than natural immunity acquired after SARS-CoV-2 infection.

After two doses of Sputnik V there is an 8.1-fold decrease in the neutralizing activity of sera against the Omicron variant, which is less than the decrease observed after two doses of other vaccines. The characteristic of Sputnik V is the use of native S glycoprotein (without proline-stabilization and other modifications that may move an immune response predominantly to the actively mutating receptor-binding domain of S glycoprotein—the Omicron variant has 16 mutations in RBD) and the use of a heterologous prime-boost vaccination regimen. This approach may allow a robust and durable immune response to be elicited after only two doses.

This study shows data on the neutralizing activity of Sputnik V-vaccinated and BNT162b2-vaccinated sera to the B.1.1.529 (Omicron) SARS-CoV-2 variant. This study has several limitations: (1) the relatively small number of tested samples in time groups, which may not reflect the dynamics of neutralizing antibodies; (2) the absence of a fixed time schedule or a follow-up design for Sputnik V-vaccinated individuals; (3) the absence of data about age-dependent and comorbidity-dependent NtAb response; (4) some samples with moderate virus-neutralizing antibody titers on Wuhan D614G variant are followed by a number of results on the Omicron variant being under the limit of detection and, hence, limited accuracy of the evaluation of the virus-neutralizing antibody titers’ decrease; (5) the heterogeneity of the vaccinated convalescents group. Nevertheless, the aim of this study was not to compare the efficacy of different vaccines in generating an antibody response able to neutralize the SARS-CoV-2 Omicron variant.

The results have shown that a two-dose vaccination regimen allows the formation of neutralizing antibodies that can neutralize the Omicron variant; whether this level of antibodies will be enough to effectively neutralize the Omicron variant to protect against infection or disease is still unclear. This study, together with previous studies, gives additional evidence for the need of a booster immunization [9,11,16,17,18]: A preliminary study suggested that the Sputnik Light booster after Sputnik V vaccination induces a robust neutralizing antibody response to the Omicron variant [11]. The immune response to the SARS-CoV-2 Omicron variant after the booster immunization is currently being actively investigated [9,11,16,17,18]. Vaccine research shows that vaccinated individuals have a significantly milder course of Omicron variant infection, and the strategy of using booster doses could allow not only the severity to be reduced, but also the frequency of COVID-19 cases caused by the Omicron variant [16]. In the scope of vaccination strategies against new SARS-CoV-2 variants, emerging evidence indicates that heterologous vaccination could improve the immune response as compared to homologous vaccination [19,20,21] with Ad26.COV2.S enhancing the immunogenicity of BNT162b2 against the Omicron variant [19].

## 5. Conclusions

Vaccination with Sputnik V allows the formation of neutralizing antibodies that can neutralize the Omicron variant.

## Data Availability

The data that support the findings of this study will be shared on reasonable request to the corresponding author.

## Supplementary Materials

The following supporting information can be downloaded at: https://www.mdpi.com/article/10.3390/vaccines10050817/s1, Table S1: Summary table of participants vaccinated with Sputnik V; Table S2: Summary table of participants vaccinated with BNT162b2 (2w, 3m, 6m); Table S3: Summary table of COVID-19 convalescent participants (samples collected at 1 to 3 months from symptom onset in march to December 2020, Italy and in January to March 2021 in Russia); Table S4: Decrease of NtAb to Omicron variant vs B.1 variant in sera of vaccinated according to basic level of RBD-specific IgG; Figure S1: RBD-specific IgG titers in vaccinated individuals, median and 95% CI. W – weeks, m – months.
