# Supplementary material for: Retention of Neutralizing Response against SARS-CoV-2 Omicron Variant in Sputnik V-Vaccinated Individuals
Source: Vaccines (Basel). 2022 May 21;10(5):817. doi: 10.3390/vaccines10050817 (PMC9144866; doi:10.3390/vaccines10050817)
Supplement: Supplementary file 1 [file vaccines-10-00817-s001.zip › vaccines-1705378-supplementary.pdf]

**Supplementary**

**Table S1**. Summary table of participants vaccinated with Sputnik V

| | Sputnik V (up to 6 months) | Sputnik V + asymptomatic* COVID-19 | Sputnik V + mild COVID-19 |
|---|---|---|---|
| Number of individuals | 31 | 8 | 12 |
| Age, years** | 33 (25-47) | 38.5 (27.25-44.75) | 36 (30.25-42.5) |
| Male sex | 16 (51.6%) | 6 (75%) | 11 (91.7%) |
| RBD-specific IgG, BAU/ml ** | 416.6 (298.8-525.9) | 878.1 (390.1-2772) | 767.2 (516.5-1459) |

* No COVID-19 in anamnesis, SARS-CoV-2 N-protein positive IgG.
** values are median (interquartile range).

**Table S2**. Summary table of participants vaccinated with BNT162b2 (2w, 3m, 6m)

| | BNT162b2 |
|---|---|
| Number of individuals | 17 |
| Age, years* | 37 (31-48) |
| Male sex | 9 (53%) |
| RBD-specific IgG, BAU/ml * | 562.7 (294.9-1640) |
| RBD-specific IgG at time point “2 weeks after vaccination”, BAU/ml * | 2326 (1466-3562) |
| RBD-specific IgG at time point “3 months after vaccination”, BAU/ml * | 562.7 (365-901.8) |
| RBD-specific IgG at time point “6 months after vaccination”, BAU/ml * | 195.2 (133.2-318.5) |

* values are median (interquartile range).

**Table S3**. Summary table of COVID-19 convalescent participants (samples collected at 1 to 3 months from symptom onset in march to December 2020, Italy and in January to March 2021 in Russia)

| | COVID-19 |
|---|---|
| Number of individuals | 23 |
| Age, years* | 42 (36-50) |
| Male sex | 13 (56.5%) |
| RBD-specific IgG, BAU/ml * | 266.5 (139.1-651.2) |

* values are median (interquartile range).

**Table S4**. Decrease of NtAb to Omicron variant vs B.1 variant in sera of vaccinated according to basic level of RBD-specific IgG

| Group | Sputnik V | | BNT162b2 | |
|---|---|---|---|---|
| | NtAb decrease, folds | % of sera with detectable NtAb | NtAb decrease, folds | % of sera with detectable NtAb |
| Total | 8.1 | 74.2 | 21.4 | 56.9 |
| 0-25% | 10.4 | 25 | 24.5 | 15.4 |
| >25-50% | 6.2 | 87.5 | 19.8 | 46.2 |
| >50-75% | 11.3 | 87.5 | 16.9 | 76.9 |
| >75-100% | 5.9 | 100 | 25.4 | 83.3 |
| 0-50% | 8.0 | 56.3 | 22.0 | 34.6 |
| >50-100% | 8.4 | 93.3 | 20.5 | 80 |
| >25-75% | 8.4 | 87.5 | 18.3 | 65.4 |

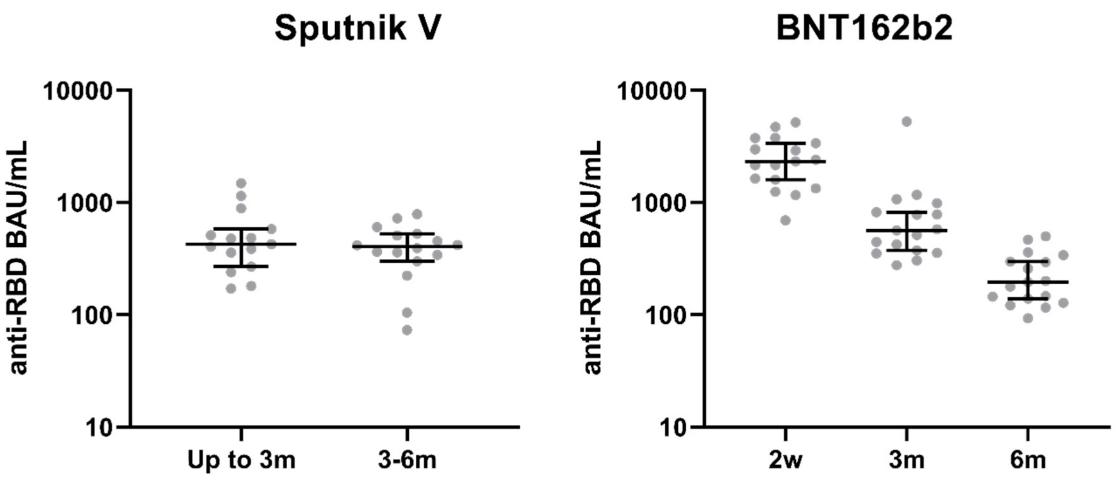


**Figure S1**. RBD-specific IgG (BAU/ml) in vaccinated individuals, median and 95% CI. W – weeks, m – months.

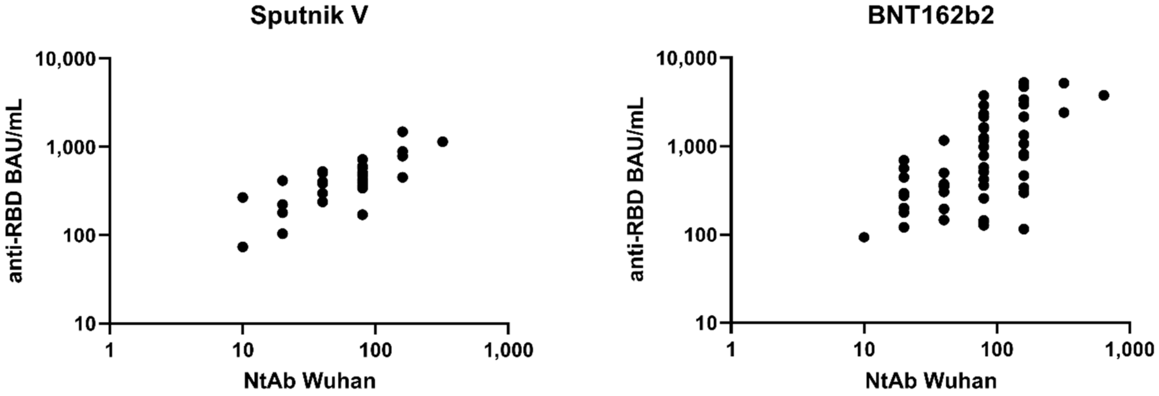


| Spearman r | |
|---|---|
| r | 0.6876 |
| 95% confidence interval | 0.4317 to 0.8410 |

| Spearman r | |
|---|---|
| r | 0.5607 |
| 95% confidence interval | 0.3299 to 0.7283 |

**Figure S2**. Correlation of RBD-specific IgG and NtAb titers in vaccinated individuals. Graphs show Spearman coefficient of correlation r and 95% CI.
